# The Effect of Atypical Nucleic Acids Structures in DNA Double Strand Break Repair: A Tale of R-loops and G-Quadruplexes

**DOI:** 10.3389/fgene.2021.742434

**Published:** 2021-10-08

**Authors:** Rosa Camarillo, Sonia Jimeno, Pablo Huertas

**Affiliations:** ^1^ Departamento de Genética, Universidad de Sevilla, Sevilla, Spain; ^2^ Centro Andaluz de Biología Molecular y Medicina Regenerativa-CABIMER, Universidad de Sevilla-CSIC-Universidad Pablo de Olavide, Sevilla, Spain

**Keywords:** R-loops, DNA double strand break repair, homologous recombination (HR), DNA end resection, G-quadruplex

## Abstract

The fine tuning of the DNA double strand break repair pathway choice relies on different regulatory layers that respond to environmental and local cues. Among them, the presence of non-canonical nucleic acids structures seems to create challenges for the repair of nearby DNA double strand breaks. In this review, we focus on the recently published effects of G-quadruplexes and R-loops on DNA end resection and homologous recombination. Finally, we hypothesized a connection between those two atypical DNA structures in inhibiting the DNA end resection step of HR.

## Non-Canonical Nucleic Acids Structures

**FIGURE 1:**
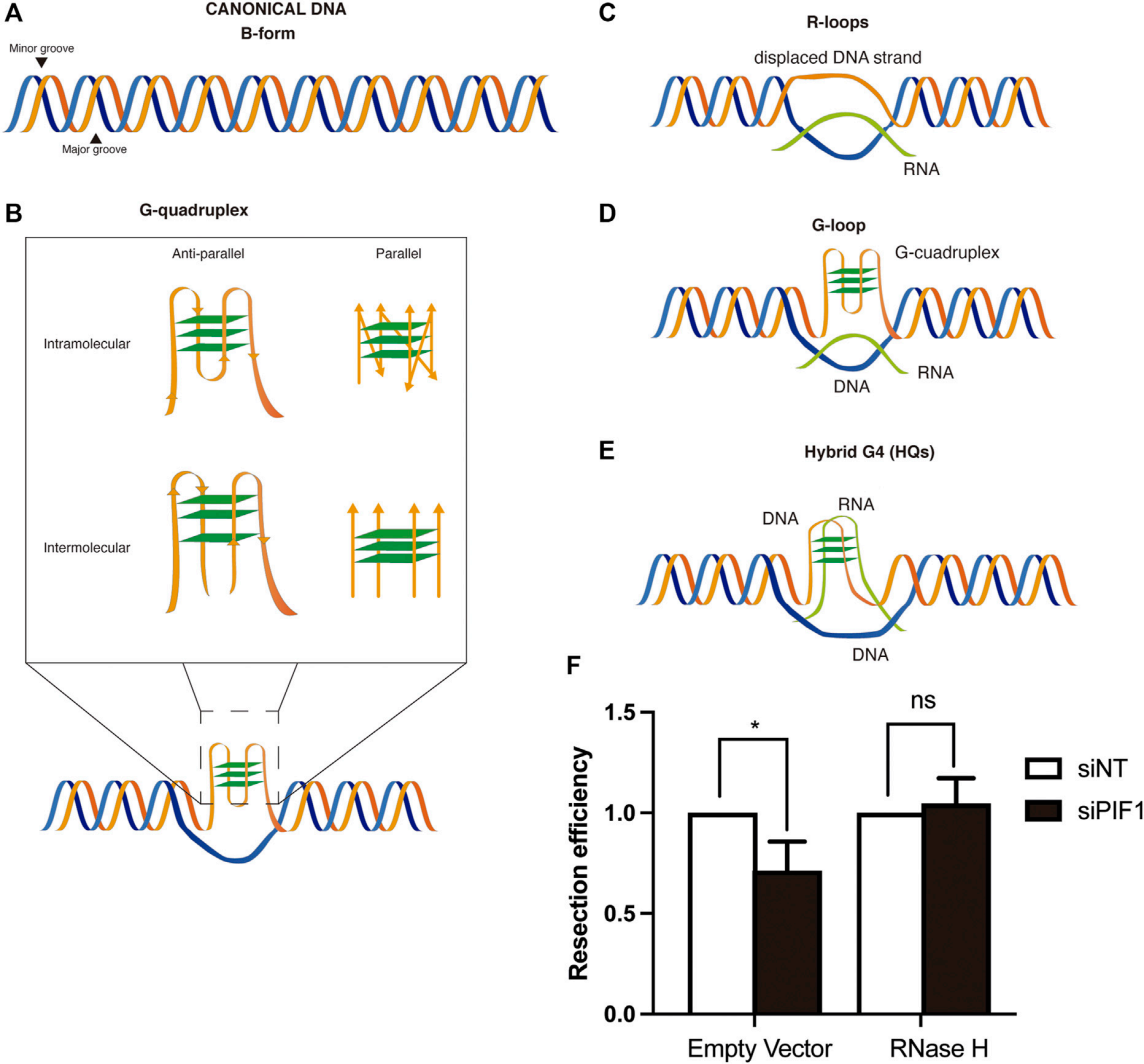
DSB repair and non-canonical DNA structures. **(A)** Canonical B-form duplex DNA structure. **(B)** Different conformations of G-quadruplexes. Inset: G-quadruplex might be intramolecular (generated on one strand of the DNA) or intermolecular (generated by several strands of DNA). In each case, they can be parallel or anti-parallel depending on the orientation of the strands. **(C)** R-loops structures are formed by the base-paired annealing of an RNA molecule with a DNA strand and the consequent displacement its complementary one. **(D)** G-loops structures arise from the formation of a G4 in the displaced ssDNA strand of an R-loop. **(E)** Hybrid G4s are chimeric structures in which the G-quadruplex is formed by the interaction of G at both a ssDNA and RNA molecules of an R-loop, displacing the other strand of the DNA **(F)** RNase H overexpression rescues the resection defect observed after PIF1 depletion. DNA resection proficiency measured as the percentage of RPA-foci-positive U2OS cells in cells expressing FLAG-RNase H or a FLAG empty vector and with either an siRNA against PIF1 (Dharmacon, CAU​AUC​UGC​UAA​AGC​GAA​U) or control siNT. Briefly, cells were seeded and grown for 24 h on coverslips. The day of transfection, medium was replaced by fresh DMEM without antibiotics and cells were incubated with a mix of siRNA and Lipofectamine diluted for 6 h in Opti-MEM before transfection with the plasmids with FuGENE six Transfection Reagent (Promega). 48 h after siRNA transfection, cells were irradiated (10 Gy) and incubated at 37°C for 1 h. Coverslips were then washed once with PBS followed by treatment with pre-extraction buffer (25 mMTris-HCl, pH 7.5, 50 mMNaCl, 1 mM EDTA, 3 mM MgCl2, 300 mM sucrose and 0.2% Triton X-100) for 5 min on ice. Cells were fixed with 4% paraformaldehyde (w/v) in PBS for 15 min. Following two washes with PBS, cells were blocked for 1 h with 5% FBS in PBS, co-stained with anti-RPA (Abcam ab2175) and anti-ϒH2AX (Cell Signaling 2,577) antibodies in blocking solution overnight at 4°C, washed again with PBS and then co-immunostained with the appropriate secondary antibodies (Alexa Fluor 594 goat anti-mouse (Invitrogen A-11032), Alexa Fluor 488 goat anti-rabbit (Invitrogen A-11034) in blocking buffer. After washing with PBS and dried with ethanol 70 and 100% washes, coverslips were mounted into glass slides using Vectashield mounting medium with DAPI (Vector Laboratories). RPA foci immunofluorescences were analyzed using a Leica Fluorescence microscope with a HCX PL APO 63x/1.4 OIL objective. In all cases, at least 200 cells were analysed per condition and the experiments were replicated independently at least three times. Significance was determined by Student’s t test comparing each condition to siNT cells. *p < 0.05.

In recent years it has been well established that DNA does not always adopt the canonical right-handed B-DNA configuration that is depicted in textbooks (Figure 1A). This DNA structure is based in two linear antiparallel DNA strands that twist together around the same axis forming a double helix that contains a major groove and a minor groove. Albeit this is the form that the most DNA acquires in the cell, there are other non-canonical conformations of DNA. Such structures have been shown to exist *in vitro* and *in vivo,* and to be related to many biological processes although their roles remain to be fully characterised. There are many non-canonical DNA structures, and exhaustive recent reviews can be found at (Kaushik et al., 2016; Saini et al., 2013). Among these alternative structures we will focus on G-quadruplexes (G4s, secondary structures arising in repetitive guanine rich areas of either DNA or RNA) (Figure 1B) (Bochman et al., 2012; Kaushik et al., 2016) and R-loops (three-stranded structures that harbour a DNA-RNA hybrid) (Figure 1C).

## G-Quadruplexes

As previously stated, G4s are non-canonical nucleic acid secondary structures formed in guanine-rich areas. The connection of four guanines by Hoogsteen hydrogen bonding generates a square planar arrangement known as G-quartet (Figure 1B). These planar G-quartets can stack on top of each other generating four-stranded helical structures. G4 structures can adopt a variety of conformations resulting from different arrangements of strand directions. Thus, G4 structures can be intramolecular (formed within one strand) or intermolecular (formed from multiple strands), parallel or antiparallel (Figure 1B) (Spiegel et al., 2020). The human genome contains thousands sequences with the potential to form such structure, known as PQS (Putative Quadruplex Sequence) (Chambers et al., 2015; Hänsel-Hertsch et al., 2016; Zheng et al., 2020). G4s are formed naturally *in vivo* (Kouzine et al., 2019; Lipps and Rhodes, 2009) but their presence can be stabilized by different drugs, known as G4 ligands (Table 1; more ligands can be found at www.g4ldb.com) (Drygin et al., 2009; Xu et al., 2017). Although G4s are widely distributed across the genome, their appearance seems to be enriched in promoters, telomeres, 5′ UTRs and splicing sites (Chambers et al., 2015). The presence of these structures in such pivotal areas for gene regulation has implicated them in a variety of biological processes.

**TABLE 1:** G-quadruplexes ligands and their main characteristics.

G-quadruplex ligand	Clinical trial	Relevant characteristics	References paper
Telomestatin	—	Telomerase inhibition	Kim et al. (2002)
		High G4s selectivity over duplex DNA	
TMPyP4	—	Telomerase inhibition	Izbicka et al. (1999)
		Poor G4s selectivity over duplex DNA	
BRACO-19	—	Reduce telomerase activity	Gowan et al. (2002)
		High G4s selectivity over duplex DNA	
RHSP4	—	Telomerase inhibition	Gowan et al. (2001)
		High G4 affinity	
CX3543/Quarfloxin	Phase I	RNA G4 affinity	Drygin et al. (2009)
		Binds ribosomal DNA G4	
CX5461	Phase I	RNA G4 affinity	Xu et al. (2017)
		Binds ribosomal DNA G4	
MM41	—	High selectivity for G4s in *BCL2* promoter	Ohnmacht et al. (2015)
Pyridostatin	—	High G4 selectivity over duplex DNA	Rodriguez et al. (2008)

More G4s ligands can be found at www.g4ldb.com

In many species, telomeric DNA consists of repetitive short G-rich sequences that fold into G4s. These structures have been implicated in the maintenance of telomeres by protecting their degradation and regulating telomere length (Zahler et al., 1991). Indeed, the use of G4 stabilizing compounds inhibits telomerase activity leading to telomere shortening (Sun et al., 1997), although not every G4 ligand is able to inhibit telomerase since this enzyme seems to be able to elongate through parallel G4s, but not antiparallel ones (Moye et al., 2015; Paudel et al., 2020; Zhang et al., 2010). The enriched presence of PQS in the promoters of transcriptionally active genes seems to highlight G4 implication in regulating gene expression (Fleming et al., 2018; Lee et al., 2020). In addition, the use of G4-ligands, such as pyridostatin (PYR) (Table 1), modulates transcription of the *BRCA1* gene in neurons (Moruno-Manchon et al., 2017). Furthermore, the deficiency of the helicase RTEL1, involved in G4 dissolution (see below), leads to an altered transcription of genes possessing potential G4 sequences in their promoters (Kotsantis et al., 2020). Also, the presence of PQS have been confirmed genome-wide at mapped replication origins of higher eukaryotes (Besnard et al., 2012; Langley et al., 2016). Notably, G-quadruplexes are of functional importance for replication origin activity since the deletion of a G-rich element known as Origin G-rich Repeated Element (OGRE) strongly reduced origin activity in mouse cells and the introduction in an ectopic origin-free area stimulated the re-establishment of a new functional origin (Prorok et al., 2019). Conversely, G4 formation also show negative effects in the normal cellular metabolism. For example*,* the presence of these structures might block DNA polymerases progression and, in consequence, collapse the replication fork (Sarkies et al., 2010). Also, as discussed below, G4s are known to promote genomic instability. Due to these negative effects, several G4 ligands (Table 1) are currently in clinical trial for cancer treatment.

## Dissolution of G4s

G4s are dynamic structures, so cells have evolved different mechanisms to resolve these structures. The main proteins associated are helicases (Mendoza et al., 2016) (see Table 2). Several helicases have been reported to be involved in G4-resolution. The main ones can be divided in several superfamilies (SF), including PIF1, BLM, WRN, RecQ, FANCJ, DNA2 and the aforementioned RTEL1 (Mendoza et al., 2016). Precisely, RTEL1 is important for the maintenance of telomeres integrity helping dismantling telomeric DNA secondary structures to allow efficient telomere replication (Vannier et al., 2012). Although those are the most studied helicases involved in this process, there has been a growth in the number of helicases with a role in G4 processing. The knock out of the helicase DHX36, for example, increases the stress response due to the stabilization of an RNA G4 (Sauer et al., 2019). Indeed, these helicases usually collaborate with other proteins to unwind G4s. That is the case of DDX1 that has been lately reported to be recruited by Timeless to the replication fork to ensure processive replication nearby G4 structures (Lerner et al., 2020).

**TABLE 2:** Helicases involved in G-quadruplexes resolution and their connection with R-loop biology.

Factor	Role in G4 biology	Mutant phenotype	R-loop related?	References
PIF1	5′-to-3′ DNA helicase, unwinds G4s and regulates telomere maintenance	Increased cancer risk	Yes	Paeschke et al. (2013)
RecQ helicases	5′-to-3′ DNA helicase, participates in DNA replication, repair and telomere maintenance	Bloom’s syndrome (BLM), Werner syndrome (WRN), Rothmund-Thomson syndrome (RECQ4)	Yes	Wu et al. (2015)
FANCJ	5′-to-3 DNA helicase, required for the repair of DNA crosslinks	Fanconi Anemia	Not determined	Wu and Spies. (2016)
DNA2	5′-to-3′ DNA helicase with nuclease activity, involved in DNA replication and DNA repair in nucleus and mitochondria	Sensitivity to DNA replication stress, genome instability, mitochondrial myopathy, and Seckel syndrome	Not determined	Masuda-Sasa et al. (2008)
RTEL1	ATP-dependent DNA helicase involved in telomere-length regulation, DNA repair and the maintenance of genomic stability	Dyskeratosis congenita and Hoyerall-Hreidarsson syndrome, telomere-related pulmonary fibrosis and/or bone marrow failure	Yes	Wu et al. (2020)
DHX36	RNA helicase, involved in genomic integrity, gene expression regulations and as a sensor to initiate antiviral responses	Aicardi-Goutieres Syndrome and Fanconi Anemia	Not determined	Sauer et al. (2019)
CHD7	Chromatin remodeling protein with DNA helicase activity	CHARGE syndrome	Not determined	Zhang et al. (2018)
EXO1	5′ → 3′ exonuclease, mediates resection at stalled forks due to G4	Telomere defects, increased fork stalling	Not determined	Stroik et al. (2020)
DDX11	5–3′ Fe–S DNA helicase, involved in DNA replication, DNA repair, heterochromatin organization and ribosomal RNA synthesis	Warsaw breakage syndrome	Not determined	Lerner et al. (2020)
DHX9	3′-to-5′ RNA helicase involved in DNA replication, transcriptional activation, post-transcriptional RNA regulation, mRNA translation and RNA-mediated gene silencing	Werner Syndrome and Abnormal Retinal Correspondence	Yes	Chakraborty and Grosse, (2011)
XPB/XPD	DNA helicase that functions in nucleotide excision repair	Xeroderma pigmentosum B/D, Cockayne’s syndrome, and trichothiodystrophy	Not determined	Gray et al. (2014)

## R-Loops

Another non-canonical secondary structure that can be formed on DNA is the R-loop. R-loops are three-stranded structures in which a DNA-RNA hybrid is formed in a dsDNA context, thus creating a displaced ssDNA region (Figure 1C). They arise usually as a consequence of negative supercoiling of the DNA template behind the transcription complex, especially in highly transcribed regions. As well as G-quadruplexes, R-loops are both implicated in physiological DNA transactions such as class switch recombination of immunoglobulins (Yu et al., 2003), telomere maintenance (Tan et al., 2020a), gene regulation (Sun et al., 2013; Grunseich et al., 2018) or in double-strand breaks (DSBs) repair (Liu et al., 2021) but also are associated with negative effects such an increase of DNA damage.

The most studied pathway in which “scheduled” R-loops have been implicated is in regulating gene expression. The enriched presence of these structures at promoters or termination regions might facilitate the modulation of this process at different steps. First, R-loops have been described to affect chromatin dynamics principally by preventing methylation of CpG islands (Ginno et al., 2012) which would favour transcription. In addition, they can also act in gene silencing since their presence correlates with higher levels of the chromatin condensation mark phosphorylated histone H3 S10 (H3S10P) (Castellano-Pozo et al., 2013). On a second level, R-loops generated from long non-coding RNA can also recruit or displace transcription regulators to the gene promoter modulating gene expression (Boque-Sastre et al., 2015; Arab et al., 2019). Finally, the presence of R-loops at the 3′end of some genes can modulate transcription termination by stalling RNAPII until resolution by RNA-DNA helicases and the nascent RNA is release and degraded (Skourti-Stathaki et al., 2014).

Although R-loops have usually been conceived as drivers of DNA damage, a new associated role in double-strand breaks (DSBs) repair is emerging for these structures. Indeed, the presence of DNA-RNA hybrids in transcriptionally active regions seems to be important for the recruitment of RAD52 and the later RAD52-dependent activity of BRCA1 in antagonizing RIF1-53BP1 blockade of DNA end resection (Yasuhara et al., 2018). The correct processing of R-loops is a key step in determining if their presence may hamper DNA repair or, on the contrary, favour the process. To regulate this, BRCA2 seems to be the responsible protein by recruiting factors involved in R-loop degradation, i. e RNase H2, and, in consequence, ensuring proper repair of the damage by Homologous Recombination (HR) (D’Alessandro et al., 2018).

The above-described beneficial roles of R-loops in DNA metabolism imply that it is not the structure *per se* but the deregulation of their processing what causes genome instability. Consistent with this idea, the mutation or loss of factors involved in R-loops resolution, such as SETX, RNase H or Fanconi Anemia (FA) factors, leads to persistent R-loops that may result in DNA damage (Wimberly et al., 2013; Herrera-Moyano et al., 2014; Cohen et al., 2018). One of the best-known pathways hampered by non-regulatory R-loops is DNA replication. The “unscheduled” presence of these structures can block replication fork progression which could result in transcription-replication collisions and, eventually, in DNA breakage (Gan et al., 2011). Furthermore, this increased rate of DNA breaks caused by R-loops has been associated with the hyper-recombination phenotype observed in a transcription impaired background that might lead to genomic instability (Huertas and Aguilera, 2003).

## G4s and R-Loops Are Connected

The similarity between both structures resides not only in their function but also in their genome distribution. In a recent genome-wide mapping, an enrichment of G4 rich regions was reported in potential R-loop forming regions (Kuznetsov et al., 2018) supporting the possibility that G4s and R-loops may act synergistically in their biological function (Lee et al., 2020; Shrestha et al., 2014), in some cases creating a novel structure that combines both and it is known as a G-loop (Duquette et al., 2004) (Figure 1D). Indeed, in a recent study, it has been observed that the formation of an R-loop at the 5’ UTR of a gene leads to the G4 folding that, in turn, stabilizes the R-loop promoting transcription (Lee et al., 2020). The current view is that the formation of an R-loop facilitates the formation of G4s in the now free ssDNA strand. Then, the formation of the G4 in turn stabilizes the R-loop structure in a positive feedback loop (Lim and Hohng, 2020). Along this lines, it has been shown that stabilization of G4s using ligands increases R-loop formation and promotes R-loop-mediated replication stress (Chappidi et al., 2020; De Magis et al., 2019; Kotsantis et al., 2020). These G-loops have been shown to have different outcomes in transcription levels depending on where is the G4 located, having a positive impact in mRNA production if it is in the non-template strand and a negative one when it is located in the template strand (Lee et al., 2020). Additionally, the possibility of the existence of hybrid G4s (HQs) formed between the nascent RNA and the coding DNA strand (that was first described in the mitochondrial R-loop (Figure 1E; Wanrooij et al., 2012)) might also be taken into account. Indeed, the co-transcriptional formation of those structures has been reported to occur and to have a role in transcription acting as a cis element of control (Zheng et al., 2013). However, and despite the inference that G-loops and HQ structures may exist *in vivo*, it has been proven technically challenging to demonstrate that they do as opposed to a simply cohabitation of G4s and R-loops in close proximity.

## G4s, R-Loops and Genomic Integrity

### The Repair of DNA Double Strand Breaks

DNA is constantly confronted by different DNA damaging sources that endanger its integrity. The most cytotoxic type of lesion are the DNA double-strand breaks (DSBs). This is because when both strands get simultaneously broken, there is not an intact template from which the DNA sequence can be restored (Bennett et al., 1993; Kawashima et al., 2017). In order to preserve genomic stability, cells have developed a well-regulated signalling cascade, known as the DNA Damage Response (DDR), to detect and repair these DNA alterations (Hosoya and Miyagawa, 2014; Majidinia and Yousefi, 2017).

In human cells, there are two main mechanisms to repair DSBs: non-homologous end joining (NHEJ) and homologous recombination (HR). On the one hand, NHEJ directly ligates the DNA ends with little processing or none at all, and functions throughout the cell cycle (Scully et al., 2019). On the other hand, HR can use an undamaged homologous DNA sequence as a template to faithfully restore the DNA sequence involved in the break (Ranjha et al., 2018; Wright et al., 2018). This pathway is only available during S and G2 phases due to the need of an identical sister chromatid to repair the broken ends.

## G4s and R-Loops Formation Increase Genomic Instability

As previously stated, R-loops are well known stimulators of genomic instability, as its presence caused an increase in replication problems and hyper-recombination (further reviewed in (García-Muse and Aguilera, 2019; Rondón and Aguilera, 2019)).

On the other hand, recently it has been described that the use of G4 ligands (Table 1) is linked to an increase in DNA damage due to the induction of replication stress at PQS (Rodriguez et al., 2012). Moreover, an increase in cell cytotoxicity is observed when G4 ligands are used in a cell background where HR-factors are impaired (Zimmer et al., 2016; Xu et al., 2017). Although their evolutionary conservation supports a physiological role of the G4-forming sequences in these DNA processes, G4 structures weaken the genome and render it prone to accumulate DNA damage when they are not efficiently regulated. Indeed, the mapping of DSBs at the genome, using the technology DSB capture, showed an enrichment of these structures nearby the DNA breaking point (Lensing et al., 2016). Also, the association of G4s with DNA damage has been usually linked to their tendency to stall replication forks and cause chromosome breakage when not properly resolved (Paeschke et al., 2011).

Additionally, when the mechanisms responsible for G4 resolution fail to do so, replication forks that encounter these structures may stall or collapse leading to DNA DSBs (Lopes et al., 2011). This situation poses an extra challenge to the cell that might employ the pathways necessary to resolve it. Since the most faithful pathway to deal with this kind of breaks is HR, it is not a surprise that this process is also implicated in G4 induced damage resolution. Notably, the stabilization of G4s in cells deficient for HR factors, such as BRCA1/2 and EXO1, leads to an increased lethality (Zimmer et al., 2016; Xu et al., 2017; Stroik et al., 2020). Furthermore, several proteins with G4 unwinding capacity are recruited to DNA damage and facilitate DNA repair. This includes various helicases involved in G4 resolution such as BLM, WRN (Bernstein et al., 2010; Croteau et al., 2014), FANCJ (Wu et al., 2008; Sarkies et al., 2012) or PIF1 (Bochman et al., 2010; Muñoz-Galván et al., 2017). Indeed, we recently demonstrated that the 5′-3′ helicase PIF1, is required for the correct resection processivity by both unwinding G4 and recruiting BRCA1 to the break (Jimeno et al., 2018).

Finally, the role of G4s in DNA repair might not be limited only to their damage inducing capacity. Indeed, in (De Magis et al., 2020), the authors showed that G4s are also positively implicated in the repair of DNA damage. This observation was supported by the fact that the stabilization of G4s by Zuo1 in *Saccharomyces cerevisiae* stimulated the recruitment of Nucleotide Excision Repair machinery acting as a loading platform which led to a more efficient repair of the damage.

### The Connection of G4s, R-Loops and Topoisomerases I and II

Recently, several studies have emerged pointing to a role of Topoisomerases in the homeostasis and function of G4s in human cells. On the one hand in a genetic CRISPR/Cas9 based multi-screen, it has been shown that both PYR and CX5461 cytotoxicity is mediated by Topoisomerase II trapping (Olivieri et al., 2020). In this study they show that DSBs produced by PYR are mainly repaired by NHEJ. This would be in accordance with the fact that this molecule negatively affects DNA end resection, a fact that would immediately bias the repair towards NHEJ (Jimeno et al., 2018). Indeed, they demonstrate that although PYR behaves different to etoposide, both increase TOP2 cleavage complexes (Olivieri et al., 2020). On the other hand, with another genetic approach, topoisomerase II-α has also been found as a major effector of the toxicity of PYR and CX-5461 clastogenic agents (Bossaert et al., 2021); they also show that, despite the stabilization of G4s after PYR treatment, DSBs accumulation needs on-going transcription. Also, a reduction in PYR-mediated DSBs is observed when RNase H1 was overexpressed. This observation is in the same line than our own data showing that RNase H1 overexpression suppresses the DNA resection phenotype observed in PIF1 mutants (Figure 1F). Both results indicate a possible role of R-loops in the DNA damage generated by G4s stabilization due to PYR. They also show that DSBs caused by G4s stabilizing agents need transcription elongation to be active in order to be formed, pointing to a role of the supercoiling produced by active transcription in the production of those DSBs. Also, in another study spontaneous DSBs have been shown to be more prone to accumulate in regions in the genome with the capacity to form stable DNA secondary structures, including G4 structures, those regions being also prone to Top2-mediated cleavage (Szlachta et al., 2020).

Indeed, a connection between G4s inducing or stabilizing agents and topoisomerases could be inferred from previous studies. For instance, topoisomerase I Inhibitors Indenoisoquinolines are able to bind and stabilize the G4 present in the promoter *of MYC* onco-gene lowering its expression (Wang et al., 2019). In another study Shuai et al. describe several short chains that can form G-quadruplex to have certain level of inhibition to topoisomerase I (Shuai et al., 2010). Also, quinolino [3,4-b]quinoxalines and pyridazino [4,3-c]quinoline derivatives showed a high activity as Topo IIα inhibitors and G-quadruplex stabilizers and also showed cytotoxic properties against two human cancer cell lines (Palluotto et al., 2016).

### R-Loops and G4s, Two Sides of the Same Coin?

As the data for a related activity between these non-canonical DNA structures arose, new evidence linking the damage induced by G4s with R-loops presence appeared. Indeed, the use of G4 stabilizers also produced an increase in R-loop formation and the suppression of these structures by RNase H1 overexpression avoided the G4-associated formation of DNA damage markers (De Magis et al., 2019). In addition, several helicases involved in G4 unwinding are also implicated in R-loop resolution (see Table 2). That is the case for BLM helicase whose deletion delayed both the clearance of G4 and R-loops with the subsequent delay in γH2AX foci, a DNA damage marker, fading (Tan et al., 2020b). Another example is the helicase DHX9 that has been shown to resolve not only R-loops but also G4s (Chakraborty and Grosse, 2011). Indeed, depletion of DHX9 reduced the levels of DNA resection (Chakraborty et al., 2018; Prados-Carvajal et al., 2018). This fact has also been demonstrated by others, since, it has been recently shown, that USP42 and DHX9 promote DSB repair, through R-loop resolution and have a role in DNA resection, as well (Matsui et al., 2020). Along the same lines, in yeast Sen1 suppresses R-loop formation at DSBs in order to promote resection and increase repair fidelity (Rawal et al., 2020). Finally, the G4 unwinding helicase PIF1, recently implicated in DNA resection (Jimeno et al., 2018), has also been reported to act on DNA-RNA hybrids by complementing RNase H activity (for review see (Pohl and Zakian, 2019)).

Interestingly, both structures are known to accumulate close to sites of DNA breaks (Bader and Bushell, 2020; Cohen et al., 2018; D’Alessandro et al., 2018; Lensing et al., 2016; Rodriguez et al., 2012). This is, in part, due to an increase propensity of DNA to break close to both structures, explaining why stimulation of their presence increase genomic instability, but at least for R-loops they seem to be formed also as a consequence of the breaks. Furthermore, treatment with some G4 ligands in cancer cell stimulate R-loop formation (Amato et al., 2020). Moreover, the increase in G4s formation or stabilization, either using PYR or by depleting PIF1, (Koirala et al., 2011; Paeschke et al., 2013; Dahan et al., 2018), or the increase of R-loops accumulation by different genetic means (García-Pichardo et al., 2017; Zhang et al., 2020; Tsai et al., 2021) both generates road-blocks for DNA resection that naturally require, in both cases at least partially, the pro-resection factor BRCA1 (Hatchi et al., 2015; Zimmer et al., 2016; Xu et al., 2017). Thus, a tantalizing hypothesis is that these two structures are connected in their effect as resection impediments, and that G4s might stimulate R-loops and/or vice versa and block resection. Along those lines, we have been able to show that RNase H1 overexpression, indeed, rescue the resection impairment phenotype observed when PIF1 is depleted (Figure 1F). Further work will be needed to clarify this hypothesis in the future.

The implication of these non-canonical secondary DNA structures in DNA damage, in the control of important processes for the biology of cancer cells and their enrichment in cancer-promoting genes has raised the possibility of using them as novel therapeutic targets. As mentioned, several G4 stabilizing compounds (Table 1) have been studied for their therapeutic potential in cancer cells deficient in proteins involved in DNA repair pathways (Xu et al., 2017; Zimmer et al., 2016). Both PYR and CX-3542 (Table 1), for example, induce an increased lethality BRCA1/2 deficient cells (Zimmer et al., 2016). Basically, when these proteins are absent, there is a deficiency in HR-based repair that leads to a failing in repairing the DSBs induced by these G4 stabilizers. In consequence, through a synthetic lethality mechanism, cells undergo apoptotic cell death. Another pathway that could be exploited for cancer therapy is the downregulation of proto-oncogenes, like c-MYC, by the stabilization of the G4s present in their promoters as studied in (Jiang et al., 2020) with the use of benzoxazinone derivatives. The effect of stabilizing these non-canonical DNA structures should also be considered in the context of a concomitant R-loop stabilization (Amato et al., 2020). This evidence clearly opens a new appealing target to discover promising new approaches in drug design for cancer chemotherapy.

## Data Availability

The raw data supporting the conclusions of this article will be made available by the authors, without undue reservation.
